# Performance Evaluation of Eco-Friendly Recycled Powder in Foamed Concrete: Influence of Powder Types and Replacement Ratios

**DOI:** 10.3390/ma18235470

**Published:** 2025-12-04

**Authors:** Xiaofang Tong, Zhiyu Zhang, Mingyi Zhang, Zhenxiang Jie, Yongfan Gong

**Affiliations:** 1 School of Civil Engineering, Yangzhou Polytechnic University, Yangzhou 225009, China; tong5150@163.com; 2 College of Civil and Transportation Engineering, Yangzhou University, Yangzhou 225127, China; 15252758817@163.com (Z.Z.); mz120241096@stu.yzu.edu.cn (M.Z.); jzxjg@foxmail.com (Z.J.)

**Keywords:** foam concrete, recycled powder, microstructure, macroscopic properties

## Abstract

The preparation of construction waste into eco-friendly recycled powder (RP), partially replacing cement to produce foam concrete with thermal insulation properties, provides a new approach for the resource utilization of RP. In this study, different components of construction waste were used to prepare recycled paste powder (RPP), recycled brick powder (RBP), and recycled concrete powder (RCP). The effects of RP on the microstructural and macroscopic properties of foam concrete were investigated at replacement rates ranging from 0% to 30%. The research results indicate that the microstructure of all three types of RP exhibits irregular shapes, and their chemical compositions show significant differences. Partial replacement of cement with these RP leads to the deterioration of the matrix microstructure, which negatively affects the workability and mechanical properties of the foam concrete. However, the addition of RP effectively mitigates the drying shrinkage of the foam concrete, with RBP showing particularly outstanding performance in this regard. Specifically, the maximum drying shrinkage rate of F-30RBP is 9.33% and 11.31% lower than that of F-30RPP and F-30RCP, respectively. Furthermore, the incorporation of RP has a minimal effect on the thermal conductivity of the foam concrete, indicating that RP is well-suited for use in foam concrete.

## 1. Introduction

With the acceleration of urbanization and the continuous update of infrastructure, the generation of construction waste has shown a sharp increase. According to statistics, the annual emission of construction waste in China has exceeded 2 billion tons, with a significant proportion of this waste being generated domestically [1,2]. Traditional methods of handling construction waste mainly involve landfilling and stockpiling in open areas. These practices not only occupy large amounts of land resources but also pose potential risks such as soil and air pollution due to heavy metal leakage and dust dispersion, which has become a major environmental issue limiting the sustainable development of cities [3,4,5]. At the same time, cement, as the core binding material in the construction industry, requires significant consumption of limestone and coal during its production process. For every ton of cement produced, approximately 0.8 tons of CO_2_ are emitted, accounting for about 7% of global industrial carbon emissions [6,7,8]. Against the backdrop of the “dual-carbon” goals and the construction of “zero-waste cities”, achieving the resource utilization of construction waste and the low-carbonization of cement-based materials has become a key research focus in the field of building materials.

Foam concrete, as a new type of building material with low density and excellent thermal insulation properties, is widely used in applications such as wall insulation, floor backfilling, and foundation pit filling, owing to its advantages of low density and low thermal conductivity [9,10,11]. However, traditional foam concrete uses cement as the primary binding material, which not only leads to higher material costs but also results in a significant carbon footprint due to the high consumption of cement [12,13]. In recent years, some researchers have attempted to replace a portion of cement with traditional supplementary cementitious materials such as fly ash [14], slag [15], and silica fume [16]. Although these materials can effectively reduce carbon emissions, fly ash and slag largely depend on industrial by-products and are geographically and structurally limited in China. Therefore, utilizing innovative recycling methods for construction waste, such as crushing and grinding it into RP [17,18,19], has emerged as a promising alternative. The RP not only has chemical compositions similar to those of supplementary cementitious materials but also contributes to both “solid waste disposal” and the development of “low-carbon building materials”. This approach offers a new research direction for the green development of foam concrete.

Barbir et al. [20] prepared concrete specimens in the laboratory and, after curing for 11 months, crushed and ground them into recycled concrete powder. Their study revealed that RCP exhibits micro-aggregate filling and nucleation effects. Wu et al. [21] found that the activity index of RCP was 75.6%. When autoclave curing was applied to enhance the reactivity of the RCP, the results showed a 13.5% increase in its activity compared to non-autoclaved samples, with the solubility of Si^4+^ reaching its maximum level [22]. Ahmed et al. [18] found that when the replacement rate of RCP was below 20%, it had a negligible effect on the mechanical strength of concrete. Similarly, Zhu et al. [23] observed that at appropriate dosages, RP contributes positively to the mechanical properties of UHPC. However, other studies have indicated that the incorporation of RBP negatively affects the mechanical properties of UHPC [24]. Chen et al. [25] explored the impact of high substitution ratios of RP on the compressive strength of foam concrete. Their findings demonstrated that as the substitution ratio increased, the compressive strength of foam concrete gradually decreased. Yao et al. [26] used RCP to replace 50-90% of cement in foam concrete and observed a similar trend in the mechanical performance. Yang et al. [27] investigated the use of RBP in foam concrete and found that when the replacement ratio of RBP was less than 15%, it had minimal effect on the compressive strength of foam concrete.

The existing literature primarily considers a single type of RP and fails to adequately address the complexity of construction waste and the specific characteristics introduced by its various components. Therefore, focusing solely on traditional RP lacks general applicability. Based on the multi-component nature of construction waste, this study prepared RPP, RBP, and RCP, and investigated the effects of these three types of RP on the performance of foam concrete at different replacement ratios. Through the analysis of the microstructure, workability, mechanical properties, and durability of foam concrete incorporating RP, this study comprehensively evaluates the impact of different types of RP on foam concrete performance. It is hoped that this research will provide new insights into the refined reuse of construction waste.

## 2. Materials and Experiments

### 2.1. Raw Materials

Waste concrete and waste brick are the primary solid wastes generated during the demolition of buildings. In addition, during the production of ready-mixed concrete, a significant amount of waste hardened cement paste is generated due to processes such as equipment cleaning, specimen preparation, and quality control. Therefore, in this study, these three types of construction waste were processed by ball milling and prepared into RPP, RBP, and RCP. In addition, the materials used in the experiment consisted of cement, fly ash, a foaming agent, and a foam stabilizer. Cement and fly ash were supplied by Yangzhou Yadong Cement Co., Ltd., Yangzhou, while the foam stabilizer, calcium stearate, was produced by Sinopharm Group, Shanghai. The foaming agent, LG-2258 type cement foaming agent, was manufactured by Shandong YouSuo Chemical Technology Co., Ltd., Linyi. All materials were sourced from China.

**Figure 1:**
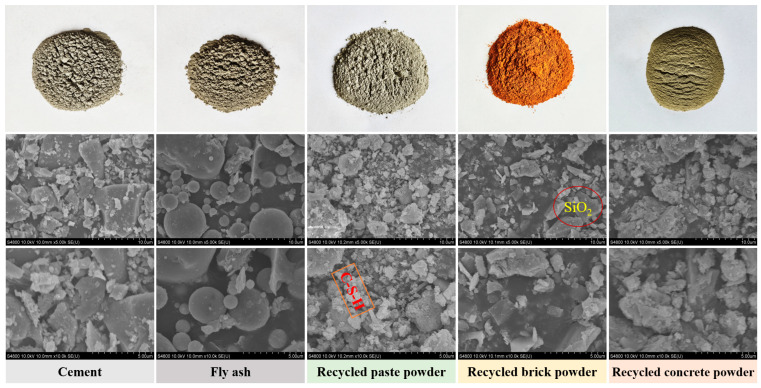
The macroscopic and microscopic morphology of cement, fly ash, and RP.

Figure 1 illustrates the macroscopic and microscopic morphology of cement, fly ash, and three different types of RP. Macroscopically, cement, fly ash, recycled paste powder (RPP), and recycled concrete powder (RCP) appear gray-black in color, while the color of recycled brick powder (RBP) is distinctly different, primarily reddish-brown. This characteristic can be attributed to the presence of iron oxides within the material. To further analyze the microstructural characteristics of the materials, this study employed SEM (produced by Hitachi, Chiyoda City, Japan, model S-4800Ⅱ) to characterize their micro-morphology, as shown in Figure 1. Fly ash exhibits a typical spherical particle morphology, while cement and the three different types of RP primarily present irregular shapes. Specifically, the microstructure of RPP and RCP shows a structure where large particles are coated with smaller particles, with hydration products such as C–S–H gel and SiO_2_ present.

**Figure 2:**
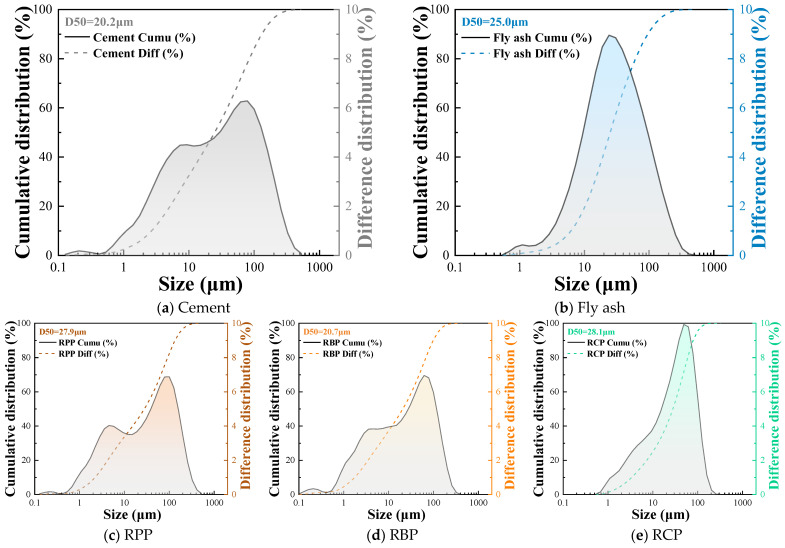
The particle size distribution of cement, fly ash, and RP.

In this study, the particle sizes of cement, fly ash, and RP were measured. From the analysis presented in Figure 2, it can be seen that the particle sizes of cement, fly ash, and RP are mostly distributed below 120 μm. Among them, the particle size distributions of cement and RBP are similar, with median diameters (D50) of 20.2 μm and 20.7 μm, respectively, indicating relatively high fineness for these two materials. In contrast, RPP and RCP have larger particle sizes, with median diameters (D50) of approximately 28.0 μm. The larger particle sizes of these materials may only serve a skeletal role in cement-based materials, leading to poorer cohesion and potentially negatively impacting the mechanical properties of the products.

**Figure 3:**
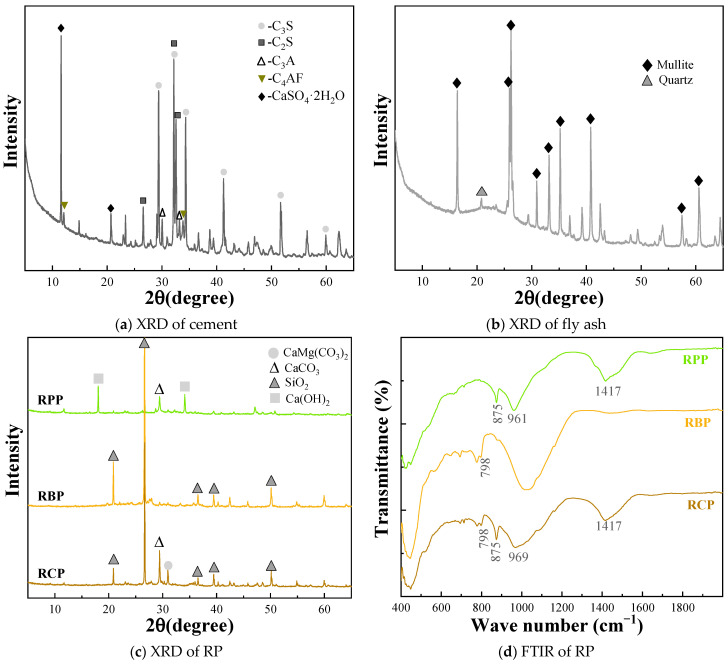
The chemical composition of cement, fly ash, and RP.

Furthermore, the chemical properties of cement, fly ash, and the three different types of RP were analyzed through, using a D8-Advance model from Bruker-AXS (Karlsruhe, Germany), and FTIR, using a Spectrum Two model from PerkinElmer (Shelton, CT, USA). As shown in Figure 3a, cement primarily consists of C_3_S, C_2_S, C_3_A, and C_4_AF, while the mineral composition of fly ash is predominantly made up of mullite and quartz. A distinct “bump peak” appears in the diffraction pattern between 20–30°, which is attributed to the presence of an amorphous phase—one of the key contributors to its reactivity [28]. RPP contains various hydration products, including Ca(OH)_2_, SiO_2_, and CaCO_3_. The main component of RBP is SiO_2_, with relatively strong diffraction peaks, indicating a high SiO_2_ content within the material. RCP consists of SiO_2_, CaCO_3_, and CaMg(CO_3_)_2_, with the presence of CaMg(CO_3_)_2_ likely originating from the coarse aggregates in the construction waste.

Additionally, FTIR analysis was conducted on the three types of RP in this study. In the RBP, a distinct absorption peak at 798 cm^−1^ corresponds to the presence of SiO_2_ [12,29], which is consistent with the XRD results. The absorption peaks at 875 cm^−1^ and 1417 cm^−1^ indicate the presence of CaCO_3_ [30]. Moreover, the absorption peak around 960 cm^−1^ represents the stretching vibration of Si–O bonds [31], indicating the presence of C–S–H gel, which is primarily derived from the hydration products of cement. These findings suggest that while the three different types of RP exhibit variations in their chemical compositions, they are generally siliceous-aluminous materials with potential for practical applications.

### 2.2. Mix Proportion and Preparation

The current work primarily investigates the effects of different types of RP and their various replacement rates on the performance of A10-grade foam concrete. Three types of RP were selected, including RPP, RBP, and RCP, which replaced cement at replacement rates of 0%, 10%, 20%, and 30% for the preparation of foam concrete. Table 1 shows the mix proportions for the recycled powder foam concrete (RPFC). Specifically, F-0RP, F-10RPP, F-20RPP, and F-30RPP represent foam concrete prepared by replacing cement with 0%, 10%, 20%, and 30% RPP, respectively; F-0RP, F-10RCP, F-20RCP, and F-30RCP represent foam concrete prepared by replacing cement with 0%, 10%, 20%, and 30% RCP, respectively; and F-10RPP, F-10RBP, and F-10RCP represent foam concrete prepared by replacing 10% of the cement with RPP, RBP, and RCP, respectively.

**Figure 4:**
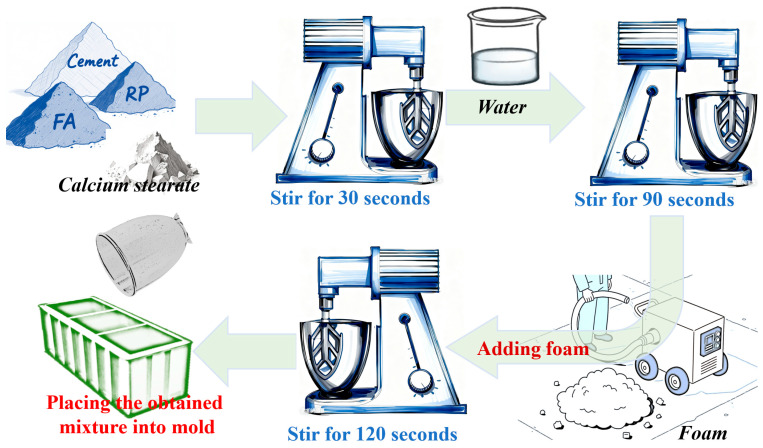
The preparation process of RPFC [12].

Figure 4 illustrates the preparation process of foam concrete. First, the cement, fly ash, RP, and calcium stearate are added to the mixing bowl according to the mix ratio presented in Table 1, and pre-mixed for 30 s. Then, water is added, and the mixture is stirred for approximately 90 s. The pre-prepared foam is quickly transferred to the mixing bowl, and stirring continues for an additional 120 s. Finally, the resulting slurry is poured into molds to form specimens, which are then covered with a plastic film and cured at room temperature for 24 h. Afterward, the specimens are demolded and transferred to a standard curing room (temperature: 20 ± 2 °C, humidity: ≥95%) for further curing until the designated test age is reached.

### 2.3. Micro-Characteristics Determination

This study will systematically characterize the hydration products (paste) using a variety of microstructural testing techniques to analyze the impact of RP on the microstructural properties of the matrix in greater depth. First, SEM will be employed to observe and analyze the microstructure of both the RP and the matrix prepared with it. Subsequently, XRD will be applied to analyze the mineral composition of the raw materials and examine the changes in the mineral composition of the paste after incorporating different types of RP, with a scanning range of 5° to 65° (2θ). Additionally, FTIR will be used to further investigate the chemical composition of the RP and the paste they produce, with a testing range from 400 cm^−1^ to 2000 cm^−1^. Furthermore, TG analysis, using a TGA 8000 model from PerkinElmer (USA), will be performed to quantitatively evaluate the effects of incorporating different types of RP on the hydration products, with testing conducted under a nitrogen atmosphere and a temperature range of 35–950 °C.

### 2.4. Workability, Thermal Conductivity and Drying Shrinkage

The flowability test in this study was conducted according to the GB/T 43487-2023 standard. Due to foam concrete exhibits excellent thermal insulation properties. Therefore, this research examines how varying RP substitution levels influence the thermal conductivity of foam concrete, with tests conducted on 30 mm × 30 mm × 300 mm thin slab specimens. The drying shrinkage properties of RPFC were tested according to the Chinese standard GB/T 43487-2023, with prism-shaped specimens measuring 40 mm × 40 mm × 160 mm used for measurement [32,33].

### 2.5. Compressive Strength, Softening Coefficient, and Carbonation Coefficient

Compressive strength is one of the key indicators for evaluating the quality of foam concrete. The compressive strength tests were conducted using cubic specimens with dimensions of 100 mm × 100 mm × 100 mm, with six specimens per group. The specimens were cured under standard conditions for 3 d and 28 d before testing, with a loading rate of 250 N/s. Additionally, the softening coefficient of foam concrete was measured to evaluate its compressive strength retention ability under saturated water absorption conditions. A total of 12 cubic specimens (100 mm × 100 mm × 100 mm) were used, with 6 specimens per group. One group of specimens was immersed in water, while the other served as the control. To further assess the durability of foam concrete, the carbonation coefficient was also tested, providing reliable experimental data to support the application of RPFC in practical engineering. The carbonation coefficient test also used 100 mm × 100 mm × 100 mm cubic specimens, totaling 24 specimens, divided into three groups: one group for carbonation testing (6 specimens), one group for observation (12 specimens), and one group for comparison (6 specimens). All macro-performance tests were conducted in accordance with the GB/T 43487-2023 standard [33].

## 3. Results and Discussion

### 3.1. Micro-Properties

**Figure 5:**
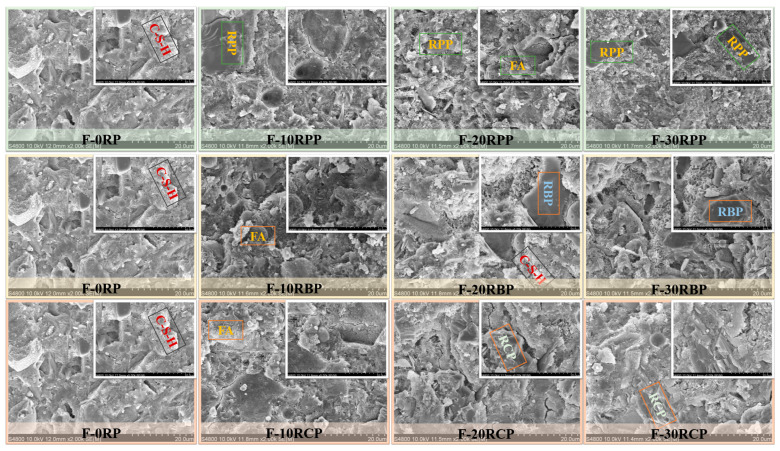
SEM results of paste containing RP.

Figure 5 shows the impact of three types of RP on the microstructure of the paste. The results show that the microstructure of F-0RP is relatively dense, with flocculent C–S–H gel forming within the matrix, indicating that cement hydration reaction is relatively complete. As RP is incorporated, the microstructure gradually shows signs of deterioration. Specifically, when the replacement rate exceeds 20%, an increase in cracks and voids is observed, and the structure becomes looser. This change may be due to the higher RP replacement rate, which reduces the cement content, causing a dilution effect that decreases the amount of hydration products, thereby weakening the density and structural integrity of the paste.

Additionally, unreacted fly ash particles are observed dispersed throughout the matrix, indicating that it has low reactivity and primarily acts as a filler, rather than directly participating in the hydration reaction. Further observation reveals that the particles of the three different types of RP are encapsulated by hydration products, with a significant amount of flocculent C–S–H gel surrounding RBP particles, indicating that RBP plays an effective microaggregate filling role within the cement matrix [34].

**Figure 6:**
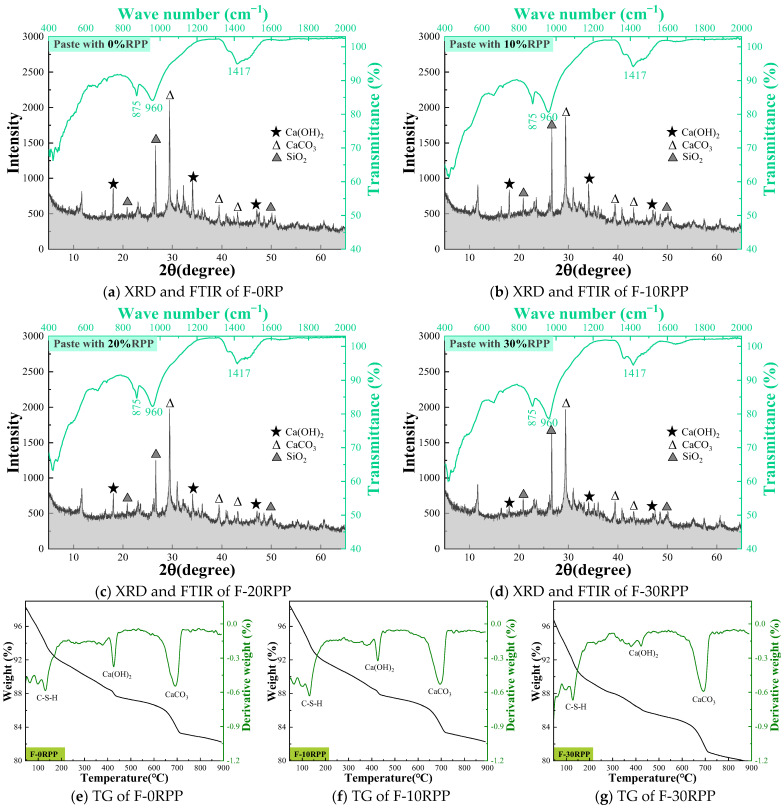
The hydration products of paste containing RPP.

Figure 6 shows the effect of different substitution ratios of RPP on the hydration products of the matrix. XRD analysis shows that the mineral composition of all groups primarily includes Ca(OH)_2_, CaCO_3_, and SiO_2_. As the RPP substitution ratio increases, the diffraction peaks of CaCO_3_ and SiO_2_ exhibit minimal changes, and the peak areas at 875 cm^−1^ and 1417 cm^−1^ in the infrared spectrum also show no significant variations, indicating that the content of CaCO_3_ remains relatively stable, consistent with the XRD analysis results. Furthermore, a distinct absorption peak appears at 960 cm^−1^, which is typically attributed to the C–S–H gel formed during cement hydration. However, the diffraction peak of Ca(OH)_2_ gradually weakens as the RPP content increases, with the diffraction peak almost completely disappearing at a 30% substitution ratio. This phenomenon suggests that the incorporation of a high proportion of RPP significantly affects the degree of hydration of the cement matrix, thereby influencing the formation of its hydration products.

TG analysis is a quantitative analytical method, and in this study, it was used to further investigate the hydration products of the matrix, as shown in Figure 6c–e. The TG curves exhibit three distinct absorption peaks, located near 100 °C, 450 °C, and 700 °C. The absorption peak around 100 °C corresponds to the dehydration of C–S–H gel, the peak near 450 °C represents the decomposition of Ca(OH)_2_, and the peak near 700 °C is associated with the decomposition of CaCO_3_ [35,36]. Notably, the changes in the area of the CaCO_3_ absorption peak for F-0RP, F-10RPP, and F-30RPP are minimal, indicating that the content of CaCO_3_ in the matrix remains relatively unchanged. In contrast, the area of the Ca(OH)_2_ absorption peak around 450 °C decreases gradually as the RPP substitution ratio increases, which is consistent with the XRD analysis results. Such behavior may be due to the reduction in cement content with increasing RPP substitution ratio, which leads to a decrease in the formation of hydration products. On the other hand, Ca(OH)_2_ reacts with the active SiO_2_ in RPP to form C–S–H gel, which further consumes Ca(OH)_2_ in the matrix [27,37].

**Figure 7:**
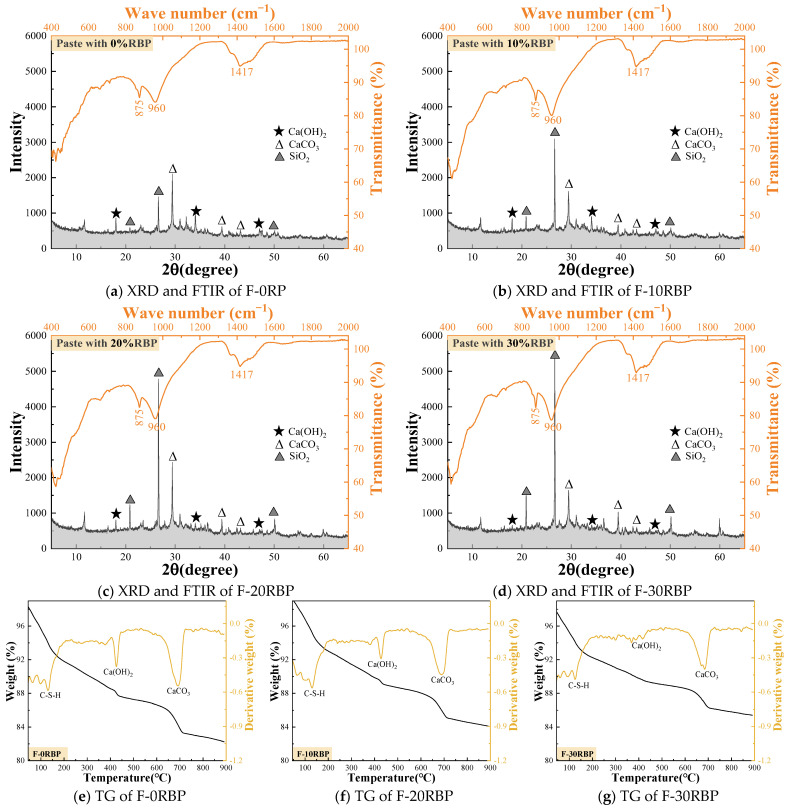
The hydration products of paste containing RBP.

Figure 7a–d show the impact of RBP substitution ratios on the hydration products of the matrix. The results indicate that the hydration products are similar to those of the RPP group, primarily consisting of Ca(OH)_2_, CaCO_3_, and SiO_2_. As the RBP substitution ratio increases, the diffraction peak of Ca(OH)_2_ gradually decreases, while the diffraction peak of SiO_2_ increases. This suggests an increase in the SiO_2_ content in the matrix, primarily due to the incorporation of RBP, which is ground from sintered clay bricks and introduces a significant amount of SiO_2_. The test results of the raw materials in Figure 1 also support this conclusion. In the infrared spectrum, the absorption peak at 960 cm^−1^ corresponds to the formation of C–S–H gel, which is further confirmed by the TG analysis. The TG curve shows a distinct absorption peak around 100 °C, indicating the presence of C–S–H gel. Meanwhile, the absorption peak around 450 °C corresponds to the formation of Ca(OH)_2_, and as the RBP substitution ratio increases, the Ca(OH)_2_ absorption peak gradually diminishes. This trend is consistent with the XRD analysis results, suggesting that the incorporation of RBP may reduce the degree of hydration of the matrix, thus affecting the formation of hydration products.

**Figure 8:**
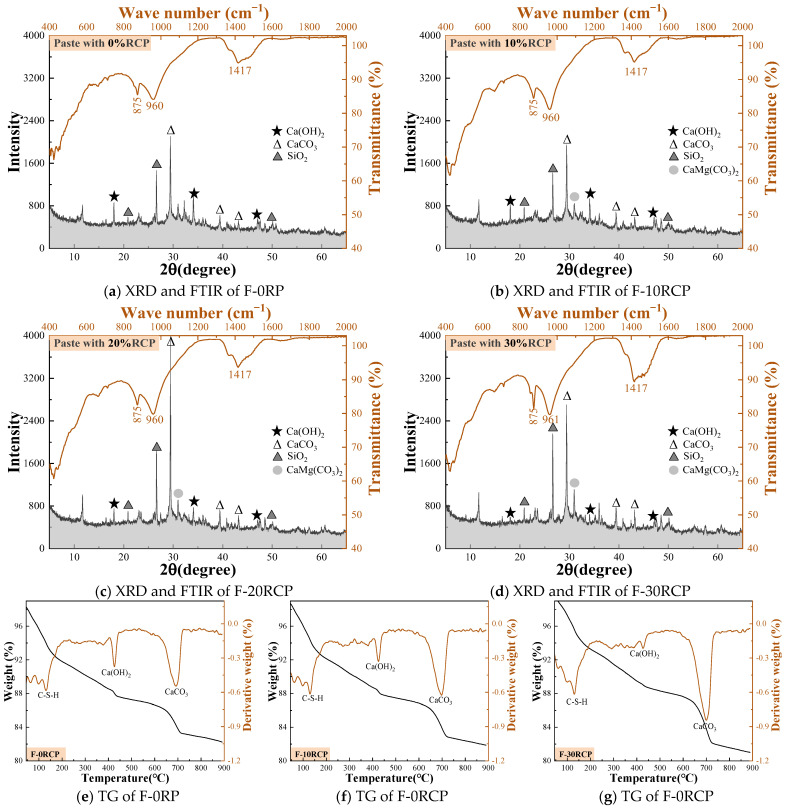
The hydration products of paste containing RCP.

Figure 8 shows the influence of RCP substitution rates on the hydration products of the matrix. Unlike the hydration products of the recycled paste powder and recycled brick powder groups, the incorporation of RCP results in the appearance of a diffraction peak for CaMg(CO_3_)_2_ in addition to the common Ca(OH)_2_, CaCO_3_, and SiO_2_. As the RCP substitution ratio increases, the intensity of this diffraction peak gradually increases, indicating a higher content of CaMg(CO_3_)_2_ in the matrix. This phenomenon is primarily due to the presence of dolomite in RCP [38], and since dolomite is difficult to participate in the hydration reaction in the cement matrix, the content of CaMg(CO_3_)_2_ in the matrix increases. Meanwhile, as RCP is incorporated, the diffraction peak of Ca(OH)_2_ in the matrix weakens, while the diffraction peaks of CaCO_3_ and SiO_2_ intensify. This change can be attributed to the high content of quartz and calcite in RCP. Furthermore, the absorption peak at 1417 cm^−1^ in the infrared spectrum increases in area with the addition of RCP, further indicating an increase in CaCO_3_ content. The TG analysis also shows that the absorption peak around 700 °C gradually increases in area, further confirming the increase in CaCO_3_ content, consistent with the XRD and FTIR results.

### 3.2. Flowability of RPFC

**Figure 9:**
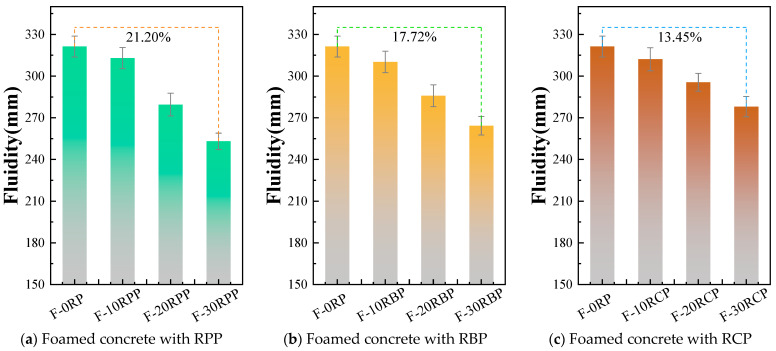
Flowability of RPFC.

Figure 9 investigates the influence of three types of RP at different substitution ratios on the flowability of RPFC paste. The results indicate that the incorporation of different types of RP leads to a reduction in paste flowability. For example, the spread of F-10RPP, F-20RPP, and F-30RPP decreased by 2.60%, 13.00%, and 21.20%, respectively, compared to F-0RP. The spread of F-10RBP, F-20RBP, and F-30RBP decreased by 3.45%, 11.02%, and 17.72%, respectively, compared to F-0RP. The spread of F-10RCP, F-20RCP, and F-30RCP decreased by 2.83%, 7.99%, and 13.45%, respectively, compared to F-0RP. Such behavior is primarily due to the irregular microstructure of the three types of RP, as shown in Figure 1, which requires more water to effectively disperse in the cement paste, thereby reducing flowability. Additionally, when the RP substitution ratio exceeds 20%, the flowability of the RPP group is poorer than that of the RBP and RCP groups. For example, the spread of F-30RPP is 4.22% and 8.95% lower than that of F-30RBP and F-30RCP, respectively.

### 3.3. Compressive Strength of RPFC

**Figure 10:**
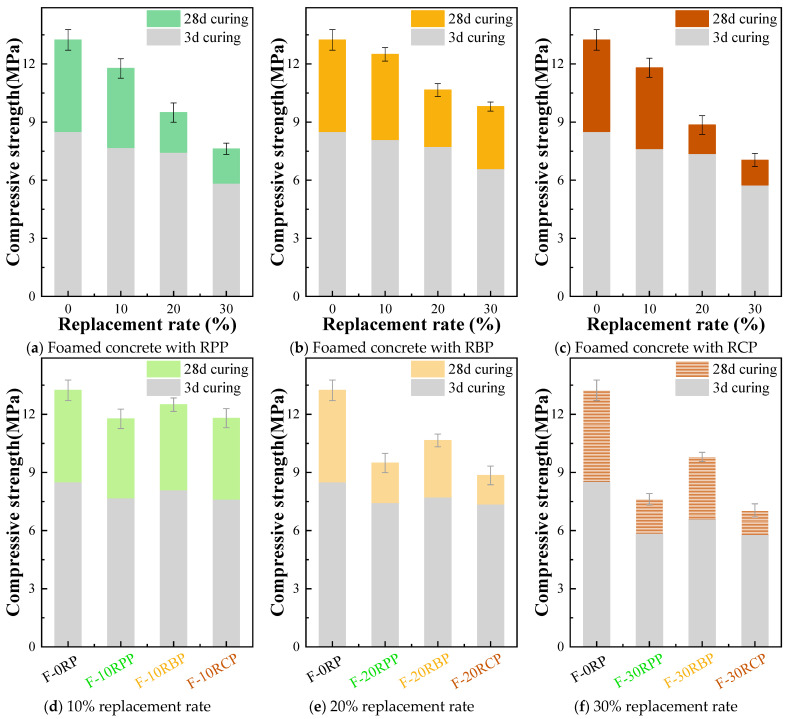
Compressive strength of RPFC.

Figure 10 shows the compressive strength of RPFC. With the prolongation of curing age, the compressive strength of RPFC gradually increases. Such as, the 28 d compressive strength of F-0RP, F-10RPP, F-20RPP, and F-30RPP increased by 55.58%, 53.06%, 27.55%, and 30.26%, respectively, compared to the 3 d compressive strength; the 28 d compressive strength of F-10RBP, F-20RBP, and F-30RBP increased by 54.32%, 37.60%, and 48.71%, respectively, compared to the 3 d compressive strength; and the 28 d compressive strength of F-10RCP, F-20RCP, and F-30RCP increased by 54.65%, 20.08%, and 22.22%, respectively, compared to the 3 d compressive strength. This increase is mainly due to the continuous hydration reactions during the curing process.

As the substitution ratio of the three types of RP increases, the compressive strength of foam concrete at 3 d and 28 d shows a gradual decrease, with the reduction in compressive strength at 28 d being more significant than at 3 d. Such as, after 3 d of curing, the compressive strength of F-10RPP, F-20RPP, and F-30RPP decreased by 9.64%, 12.57%, and 31.26%, respectively, compared to F-0RP; the compressive strength of F-10RBP, F-20RBP, and F-30RBP decreased by 4.82%, 9.05%, and 22.56%, respectively, compared to F-0RP; and the compressive strength of F-10RCP, F-20RCP, and F-30RCP decreased by 10.34%, 13.40%, and 32.31%, respectively, compared to F-0RP. When the curing age reaches 28 d, the compressive strength of F-10RPP, F-20RPP, and F-30RPP decreases by 11.10%, 28.32%, and 42.45%, respectively, compared to F-0RP; the compressive strength of F-10RBP, F-20RBP, and F-30RBP decreases by 5.59%, 19.56%, and 25.98%, respectively, compared to F-0RP; and the compressive strength of F-10RCP, F-20RCP, and F-30RCP decreases by 10.88%, 33.16%, and 46.83%, respectively, compared to F-0RP.

Such behavior is primarily attributed to the increase in RP content, which reduces the amount of materials available for sustained hydration reactions within the system. As a result, the quantity of hydration products generated in the later stages decreases, thus slowing the growth of compressive strength. The earlier microstructural analysis also indicated that the incorporation of RP results in a reduction in the generation of hydration products, which degrades the microstructure of the matrix (as shown in Figure 5), thereby negatively affecting the compressive strength of foam concrete. The XRD, FTIR, and TG test results further confirm that with increasing RP content, the formation of hydration products such as Ca(OH)_2_ is reduced [39], providing additional microscopic evidence for the adverse effect on the compressive strength.

Figure 10d,e compare the effects of different types of RP on the compressive strength of foam concrete. The findings show that at 3 d of curing, the type of RP has a minimal impact on the compressive strength of foam concrete. For example, the compressive strengths of F-20RPP, F-20RCP, and F-20RBP are 7.69 MPa, 8.10 MPa, and 7.63 MPa, respectively. However, at 28 d of curing, the RBP group demonstrates better compressive strength. Specifically, the compressive strength of F-10RBP is 6.20% and 5.93% higher than that of F-10RPP and F-10RCP, respectively; the compressive strength of F-20RBP is 12.22% and 20.34% higher than that of F-20RPP and F-20RCP, respectively; and the compressive strength of F-30RBP is 28.61% and 39.20% higher than that of F-30RPP and F-30RCP, respectively. This may be due to the similarity in particle size between RBP and cement particles, which allows RBP to better exert the microaggregate filling effect, thus improving the compactness of foam concrete. Furthermore, compared to RPP and RCP, RBP contains more SiO_2_ and alumino-silicate components, which provide higher reactivity in the cement matrix, promoting hydration reactions and ultimately enhancing strength.

### 3.4. Carbonation Coefficient and Softening Coefficient of RPFC

**Figure 11:**
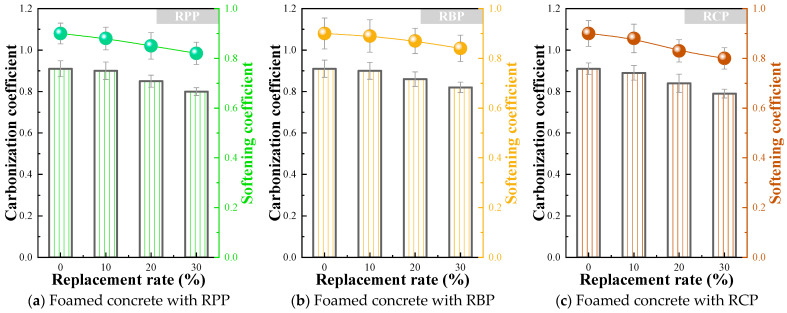
Carbonation coefficient and softening coefficient of RPFC.

The carbonation coefficient is a key indicator for assessing the resistance of foam concrete to CO_2_ erosion from the atmosphere, and its measurement aims to evaluate the long-term strength retention of the material. The carbonation specimens and observation specimens were dried in a (65 ± 2) °C drying oven for 48 h, then cooled in a desiccator before being placed in a carbonation test chamber. A separate group of control specimens was kept under standard testing conditions. After 7 d of carbonation, one observation specimen was removed every 2 d, split, and the carbonation depth was measured using phenolphthalein ethanol solution. If no red coloration appeared at the center of the cross-section, it was determined to be fully carbonated. If red coloration was observed, the specimen was discarded and testing continued. The test was concluded once the specimen had either fully carbonated or reached 28 d without complete carbonation. The final comparison of compressive strength was made between the fully carbonated specimens or those carbonated for 28 d and the control specimens, as shown in Figure 11. The results show that the incorporation of different types of RP negatively affects the carbonation coefficient, and as the substitution ratio increases, the carbonation coefficient gradually decreases. For example, the carbonation coefficients of F-10RPP, F-20RPP, and F-30RPP are 1.10%, 6.59%, and 12.09% lower than that of F-0RP, respectively. The carbonation coefficients of F-10RBP, F-20RBP, and F-30RBP are 1.10%, 5.49%, and 9.89% lower than that of F-0RP, respectively. The carbonation coefficients of F-10RCP, F-20RCP, and F-30RCP are 2.20%, 7.69%, and 13.19% lower than that of F-0RP, respectively. Overall, the reduction in the carbonation coefficient is relatively small, which may be attributed to the decreased Ca(OH)_2_ content in the matrix after the incorporation of RP (as shown in Figure 6, Figure 7 and Figure 8), leading to fewer substances available for reaction with CO_2_ in the air, thus limiting the progression of the carbonation reaction.

This study also characterized the effect of different types of RP on the softening coefficient of foam concrete. The softening coefficient is an indicator used to assess the strength retention of RPFC after water absorption, and it is primarily employed to evaluate the material’s adaptability and safety in humid environments. The water-immersed specimens were placed at the bottom of a constant-temperature water tank, while a separate group of control specimens was kept under standard testing conditions. Water was added in three stages: the first stage to one-third of the specimen height, maintaining it for 24 h; the second stage to two-thirds of the specimen height, also for 24 h; and the third stage, with the water level raised 25 mm above the specimen, maintained for 120 h. After the test, the specimens were removed, surface moisture was wiped off, and the compressive strength of the water-immersed specimens was compared with that of the control specimens. The test results are shown in Figure 11. The results indicate that, as the RP substitution ratio increases, the softening coefficient of foam concrete gradually decreases. Upon comparing the foam concrete with different types of RP, it was found that the RBP group exhibited a higher softening coefficient than the RPP and RCP groups. For example, the softening coefficient of F-30RBP was 2.44% and 5.00% higher than that of F-30RPP and F-30RCP, respectively.

### 3.5. Drying Shrinkage of RPFC

**Figure 12:**
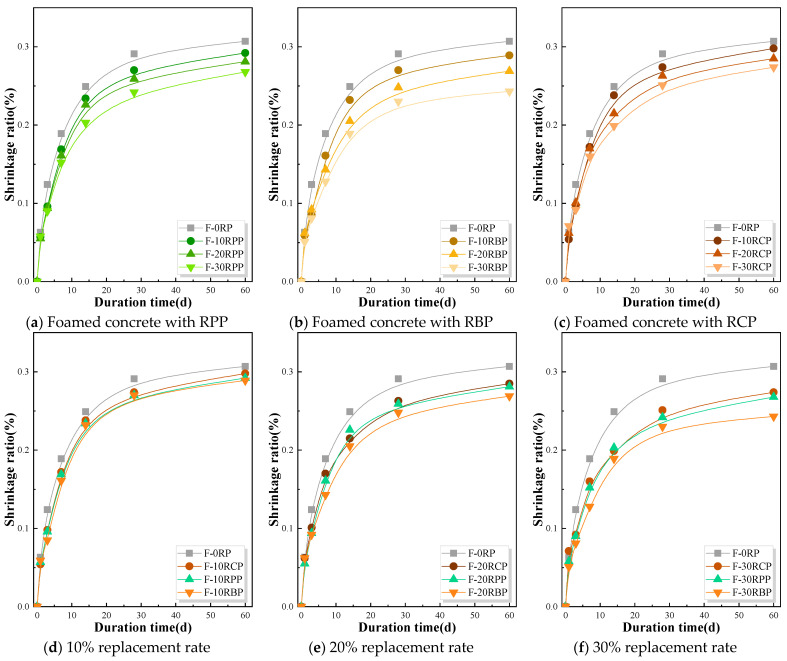
Drying shrinkage of RPFC.

Due to its high porosity, foam concrete exhibits greater shrinkage compared to ordinary concrete. Therefore, this section explores the impact of different types and substitution ratios of RP on the drying shrinkage of foam concrete. First, the specimens were placed in a constant-temperature water bath at (20 ± 2) °C. After 72 h, they were removed from the water and the surface was wiped clean. The initial length of the foam concrete specimens was measured using a length comparator. The specimens were then placed in a drying shrinkage chamber with a temperature of (20 ± 2) °C and relative humidity of (60 ± 5)%. The length changes in the specimens were measured at 1, 3, 7, 14, 28, and 60 d. The drying shrinkage rate was calculated based on these measurements, and the results are shown in Figure 12. The findings show that the drying shrinkage rate of RPFC rises quickly at first, before stabilizing over time. During the first 28 d of testing, the drying shrinkage of RPFC increases quickly. For example, the 28 d drying shrinkage rates of F-0RP, F-10RPP, F-20RPP, and F-30RPP reached 94.79%, 92.47%, 92.17%, and 90.30% of their maximum values, respectively; the 28 d drying shrinkage rates of F-10RBP, F-20RBP, and F-30RBP reached 93.43%, 92.19%, and 94.65%, respectively; and the 28 d drying shrinkage rates of F-10RCP, F-20RCP, and F-30RCP reached 91.95%, 92.28%, and 91.61%, respectively. However, as the testing period increased, the drying shrinkage rate of RPFC significantly slowed down and stabilized.

Furthermore, all three types of RP effectively suppressed the drying shrinkage of foam concrete, and with the increase in the RP substitution ratio, the drying shrinkage rate gradually decreased. Such as, the 60 d drying shrinkage rates of F-10RPP, F-20RPP, and F-30RPP were reduced by 4.89%, 8.47%, and 12.70%, respectively, relative to F-0RP; the 60 d drying shrinkage rates of F-10RBP, F-20RBP, and F-30RBP were reduced by 5.86%, 12.38%, and 20.85%, respectively, relative to F-0RP; and the 60 d drying shrinkage rates of F-10RCP, F-20RCP, and F-30RCP were reduced by 2.93%, 7.17%, and 10.75%, respectively, relative to F-0RP. This is primarily because RP contains significant amounts of inert materials (as shown in Figure 2), which remain unreacted during the early hydration of the cement-based components. Therefore, the addition of RP reduces the degree of hydration in the system, thus slowing down the occurrence of drying shrinkage [32,40]. This indicates that an appropriate amount of RP incorporation can improve the shrinkage performance of foam concrete to some extent.

This study also compared the impact of different types of RP on the drying shrinkage of foam concrete at the same RP substitution ratio, as shown in Figure 12. The analysis indicates that the drying shrinkage rates of the RPP group and the RCP group are quite similar, while the RBP group exhibits relatively lower drying shrinkage rates. For example, the 60 d shrinkage rate of F-10RBP is 3.02% and 1.03% lower than that of F-10RPP and F-10RCP, respectively; the 60 d drying shrinkage rate of F-20RBP is 5.61% and 4.27% lower than that of F-20RPP and F-20RCP, respectively; and the 60 d drying shrinkage rate of F-30RBP is 11.31% and 9.33% lower than that of F-30RPP and F-30RCP, respectively. It is worth noting that when the RP substitution ratio attains 30%, the differences in drying shrinkage rates between the different types of RP are significantly magnified. This is mainly because RBP, compared to RPP and RCP, exhibits better pozzolanic and micro-aggregate filling effects [41], which effectively improves the drying shrinkage performance of foam concrete.

### 3.6. Thermal Conductivity of RPFC

The thermal conductivity of foam concrete is an important indicator for quantifying its heat transfer capacity and evaluating its thermal insulation performance. This property directly determines whether foam concrete can meet building energy-saving requirements and is a core evaluation criterion for its application as an insulating material. Therefore, this study examines the impact of different types of RP on the thermal conductivity of foam concrete. The specimens were placed in an oven and dried to a constant weight, then allowed to cool to room temperature. Two specimens were used per group, and the thermal conductivity was measured using a DRXS3030 thermal conductivity tester. 

**Figure 13:**
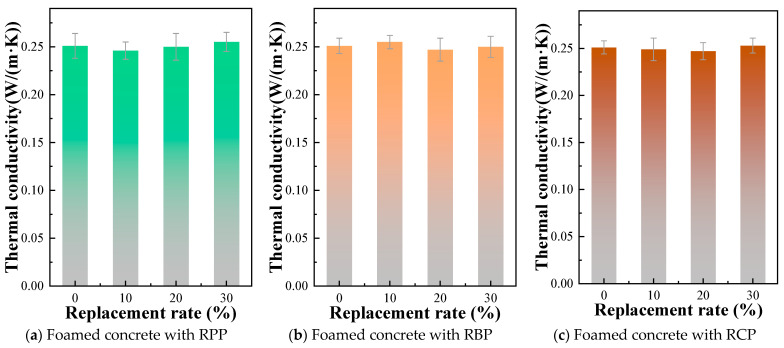
Thermal conductivity of RPFC.

Figure 13 presents the thermal conductivity of RPFC. The results indicate that the thermal conductivity of foam concrete changes only slightly with the incorporation of different types of RP. For example, the thermal conductivity of F-0RP, F-10RPP, F-20RPP, and F-30RPP were 0.251 W/(m·K), 0.246 W/(m·K), 0.250 W/(m·K), and 0.255 W/(m·K), respectively; the thermal conductivity of F-10RBP, F-20RBP, and F-30RBP were 0.255 W/(m·K), 0.247 W/(m·K), and 0.250 W/(m·K); the thermal conductivity of F-10RCP, F-20RCP, and F-30RCP were 0.249 W/(m·K), 0.247 W/(m·K), and 0.253 W/(m·K), respectively. The above results indicate that the incorporation of RP did not significantly affect the thermal conductivity of foam concrete, suggesting that different types of RP are well-suited for use in foam concrete without compromising its thermal insulation performance. This provides strong support for the application of foam concrete in building insulation.

Table 2 presents the macroscopic properties of RPFC. Analysis of the experimental data reveals that when the replacement ratio of the three types of RP is 10%, their effect on the workability and mechanical properties of foam concrete is minimal. Furthermore, the incorporation of RP effectively mitigates the drying shrinkage of foam concrete, with the RBP showing the most significant effect in reducing drying shrinkage. The addition of RP has little impact on the thermal conductivity of foam concrete. Based on the experimental results, this study recommends an optimal RP content of 10%.

## 4. Conclusions

This study examined the fundamental characteristics of various types of RP and employed them as partial substitutes for cement in the production of foam concrete. The study further explored the impact of RP substitution ratio on the microstructural and macroscopic properties of foam concrete. Based on the research findings, the following conclusions were drawn:

1. Three types of RP all exhibit irregular microstructures, but their chemical compositions differ significantly. For example, RPP contains Ca(OH)_2_, RBP contains a large amount of SiO_2_, and RCP contains CaMg(CO_3_)_2_. When these powders are partially substituted for cement, the matrix’s microstructure progressively exhibits signs of degradation. XRD and TG results indicate that, with increasing RP substitution ratios, the amount of Ca(OH)_2_ in the matrix gradually decreases.
2. Different types of RP have a negative impact on the flowability and mechanical properties of foam concrete. With the increase in the RP substitution ratio, the compressive strength of the foam concrete gradually decreases. The decline in mechanical properties is particularly significant when the RP substitution ratio reaches 30%. However, at the same substitution ratio, the recycled brick powder group shows better compressive strength. For example, the 28 d compressive strength of F-30RBP is 28.61% and 39.20% higher than that of F-30RPP and F-30RCP, respectively.
3. The incorporation of all three types of RP contributes to the suppression of drying shrinkage in foam concrete, with the drying shrinkage gradually decreasing when the RP substitution ratio increases. Notably, the RBP group demonstrates superior performance in inhibiting the drying shrinkage of foam concrete.
4. As the substitution ratio of the three types of RP increases, both the carbonation coefficient and the softening coefficient of RPFC show a decreasing trend. Furthermore, the incorporation of RP has a minimal effect on the thermal conductivity of RPFC, indicating its good applicability in foam concrete.
5. Although this study systematically investigates the microstructural and macroscopic properties of foam concrete prepared with different types of RP, research on the pore structure remains limited. Future studies will focus on the rheological properties of foam concrete slurry, aiming to further optimize its pore structure and achieve precise control over the performance of foam concrete.

## Figures and Tables

**Table 1:** Mix proportion of RPFC (kg/m^3^).

	Cement/kg	Fly Ash/kg	RPP/kg	RBP/kg	RCP/kg	Water/kg	Calcium Stearate/kg	Foam/m^3^
F-0RP	720.45	80.05	0	0	0	400.25	6.00	0.508
F-10RPP	648.41	80.05	72.04	0	0	400.25	6.00	0.508
F-20RPP	576.36	80.05	144.09	0	0	400.25	6.00	0.508
F-30RPP	504.31	80.05	216.14	0	0	400.25	6.00	0.508
F-10RBP	648.41	80.05	0	72.04	0	400.25	6.00	0.508
F-20RBP	576.36	80.05	0	144.09	0	400.25	6.00	0.508
F-30RBP	504.31	80.05	0	216.14	0	400.25	6.00	0.508
F-10RCP	648.41	80.05	0	0	72.04	400.25	6.00	0.508
F-20RCP	576.36	80.05	0	0	144.09	400.25	6.00	0.508
F-30RCP	504.31	80.05	0	0	216.14	400.25	6.00	0.508

**Table 2:** Comparison of macroscopic properties of RPFC.

	Flowability (mm)	Compressive Strength (MPa)	Carbonation Coefficient	Softening Coefficient	Drying Shrinkage (%)	Thermal Conductivity (W/(m·K))
F-0RP	321.33	13.24	0.91	0.90	0.307	0.251
F-10RPP	312.99	11.77	0.90	0.88	0.292	0.246
F-20RPP	279.56	9.49	0.85	0.85	0.281	0.250
F-30RPP	253.21	7.62	0.80	0.82	0.268	0.255
F-10RBP	310.25	12.50	0.90	0.89	0.289	0.255
F-20RBP	285.92	10.65	0.86	0.87	0.269	0.247
F-30RBP	264.38	9.80	0.82	0.84	0.243	0.250
F-10RCP	312.25	11.80	0.89	0.88	0.298	0.249
F-20RCP	295.64	8.85	0.84	0.83	0.285	0.247
F-30RCP	278.11	7.04	0.79	0.80	0.274	0.253

## Data Availability

The original contributions presented in the study are included in the article. Further inquiries can be directed to the corresponding author.
